# Rapid Affinity Maturation of Novel Anti-PD-L1 Antibodies by a Fast Drop of the Antigen Concentration and FACS Selection of Yeast Libraries

**DOI:** 10.1155/2019/6051870

**Published:** 2019-12-28

**Authors:** Biancamaria Cembrola, Valentino Ruzza, Fulvia Troise, Maria Luisa Esposito, Emanuele Sasso, Valeria Cafaro, Margherita Passariello, Feliciano Visconte, Maddalena Raia, Luigi Del Vecchio, Anna Morena D'Alise, Riccardo Cortese, Elisa Scarselli, Nicola Zambrano, Claudia De Lorenzo, Alfredo Nicosia

**Affiliations:** ^1^Department of Molecular Medicine and Medical Biotechnology, University of Naples Federico II, Via S. Pansini 5, 80131 Naples, Italy; ^2^CEINGE–Biotecnologie Avanzate s.c.a r.l., Via G. Salvatore 486, 80145 Naples, Italy; ^3^Nouscom s.r.l., Via di Castel Romano 100, 00128 Rome, Italy; ^4^Reithera s.r.l., Via di Castel Romano 100, 00128 Rome, Italy; ^5^Department of Biology, University of Naples Federico II, Cupa Nuova Cintia 21, 80126 Naples, Italy

## Abstract

The affinity engineering is a key step to increase the efficacy of therapeutic monoclonal antibodies and yeast surface display is the most widely used and powerful affinity maturation approach, achieving picomolar binding affinities. In this study, we provide an optimization of the yeast surface display methodology, applied to the generation of potentially therapeutic high affinity antibodies targeting the immune checkpoint PD-L1. In this approach, we coupled a 10-cycle error-prone mutagenesis of heavy chain complementarity determining region 3 of an anti‐PD-L1 scFv, previously identified by phage display, with high-throughput sequencing, to generate scFv-yeast libraries with high mutant frequency and diversity. In addition, we set up a novel, faster and effective selection scheme by fluorescence-activated cell sorting, based on a fast drop of the antigen concentration between the first and the last selection cycles, unlike the gradual decrease typical of current selection protocols. In this way we isolated 6 enriched mutated scFv-yeast clones overall, showing an affinity improvement for soluble PD-L1 protein compared to the parental scFv. As a proof of the potency of the novel approach, we confirmed that the antibodies converted from all the mutated scFvs retained the affinity improvement. Remarkably, the best PD-L1 binder among them also bound with a higher affinity to PD-L1 expressed in its native conformation on human-activated lymphocytes, and it was able to stimulate lymphocyte proliferation *in vitro* more efficiently than its parental antibody. This optimized technology, besides the identification of a new potential checkpoint inhibitor, provides a tool for the quick isolation of high affinity binders.

## 1. Introduction

Monoclonal antibodies (mAbs) are widely used as therapeutics for various types of disorders, such as autoimmune diseases [1], infectious diseases [2], post‐transplantation immunosuppressive regimens [3] and cancer [4]. The market of mAbs is in constant increase, with 82 mAbs approved by the Food and Drug Administration (FDA) to date and hundreds being in clinical trials [5, 6]. In parallel with the discovery of novel therapeutic mAbs, the field of antibody engineering is in constant development too, in order to improve various antibody properties for more effective therapies [7, 8]. Of main importance is the affinity engineering, which contributes to increase binding selectivity too. This aspect is of particular relevance in cancer treatment, where the selective targeting of tumor cells reduces the risk of side effects associated with conventional chemotherapy, sparing healthy cells. Moreover, high affinity antibodies ensure an improvement of therapeutic regimens, allowing for a reduction in the dosage or in the number and frequency of administrations.

The affinity maturation technologies mimic the *in vivo* antibody maturation occurring in B cells during the immune response [9–11], but achieving higher affinities (from 10^−10^ to 10^−15^ mol/L) than those obtained *in vivo* (about 10^−10^ mol/L) [12–14]. These technologies are based on random mutation of the antibody binding sites [15–20] and the subsequent selection of the antibody variants showing the highest affinity for the target, using a variety of display methods (yeast surface display, phage display, *E*. *coli* surface display, mammalian cell display, ribosome display and mRNA display) [21–23]. Among them, yeast surface display (YSD) is the most widely used affinity maturation platform because it combines a lot of advantages compared with the other approaches [24–27]; in particular, the eukaryotic machinery ensures the correct folding and post‐translational modifications of the displayed proteins; in addition, the *in vivo* yeast recombination is more efficient than *in vitro* cloning by ligation for the library generation [28]; more importantly, above all, clones with improved affinity can be selected by fluorescence-activated cell sorting (FACS). This allows a real-time quantification of both the protein display level and the antigen-binding strength directly during the screening process, discriminating even little differences in the binding properties of the antibody variants. Three to five sequential sortings with increasing selection stringency (i.e., lower and lower antigen concentrations at each selection step) are generally required for the isolation and enrichment of the yeasts with better antigen binding capacity compared with that of the parental antibody [24–26].

Among all the FDA-approved mAbs, many are used for cancer treatment, as “naked antibodies” or immunoconjugates, aiming at the direct targeting and destruction of cancer cells [29]. However, a deeper understanding of cancer biology has brought to light that tumor cells have developed a lot of strategies to block the immune surveillance, resulting in tumor growth and progression and thus explaining the resistance to conventional anticancer treatments [30–36]. For this reason, an innovative antibody-based therapeutic approach has emerged, whose purpose is to “awaken” the patient's immune system to fight cancer.

Among the mechanisms developed by tumors to escape the immune surveillance, one that aroused a great interest is the upregulation of molecules that activate the immune checkpoints [37], which are responsible for the suppression of T cell functions. The programmed death-1 receptor (PD-1) pathway is the best characterized and the most targeted immune checkpoint [38, 39]. In fact, its ligand programmed death ligand-1 (PD-L1) is frequently overexpressed in many kinds of hematologic and solid tumors [40–42], as well as on the cells of the tumor stroma [43, 44], making them resistant to the T cell-mediated killing. For this reason, a lot of mAbs have been designed to target the PD-1/PD-L1 pathway. Pembrolizumab and nivolumab are the two anti‐PD-1 mAbs approved for clinics to date, but many others are in clinical trials. Mainly developed to overcome resistance and toxicity associated with the anti‐cytotoxic T lymphocyte-associated antigen 4 (CTLA-4) ipilimumab (the first checkpoint inhibitor approved by the FDA [45]), they were firstly used for metastatic melanoma [46, 47], but they have been recently approved for the treatment of several other tumors, such as non-small cell lung cancer (NSCLC), head and neck carcinoma, renal cell carcinoma (RCC), Hodgkin's lymphoma, and squamous cell lung cancer [48–51]. Moreover, they are also under evaluation in combination regimens with chemotherapy or other checkpoint inhibitors [52–54]. A similar scenario is for the anti‐PD-L1 mAbs, with three approved PD-L1 inhibitors, that is, atezolizumab for urothelial carcinoma and non-small cell lung cancer [55, 56], avelumab for metastatic Merkel-cell carcinoma [57] and durvalumab for urothelial carcinoma and unresectable non-small cell lung cancer [58, 59].

Despite the success of the immune checkpoint blockade, there are still many patients that do not respond or relapse after treatment and others that show mild to severe adverse effects. To overcome these limitations, there is an increasing interest in discovering new checkpoint inhibitors, to plan alternative treatments or combination regimens with the already available antibodies, to produce synergistic effects [60, 61].

Our research group identified by phage display a novel, fully human anti‐PD-L1 antibody with low nanomolar apparent affinity for PD-L1; this mAb was able to induce T cell proliferation *in vitro* and showed *in vivo* antitumor activity [62]. In this work, we generated novel anti‐PD-L1 single chain variable fragments (scFvs) by affinity maturation of heavy chain complementarity determining region 3 (HCDR3) of the mAb previously characterized, using yeast surface display. In particular, we proposed a faster and more effective affinity maturation approach, through an optimization of both library generation and screening steps, to achieve a rapid isolation of mutants with improved affinity. These antibody fragments could be very good candidates for a PD-L1-targeted cancer immunotherapy because they retained the affinity improvement when converted into full length antibodies and they were able to stimulate lymphocyte proliferation *in vitro* more efficiently than the parental mAb.

## 2. Materials and Methods

### 2.1. Cell Cultures

The human embryonic kidney EBNA 293 cells (Invitrogen, Thermo Fisher, Waltham, Massachusetts, USA) were grown in Dulbecco's modified Eagle's medium (Gibco, Thermo Fisher Scientific, Waltham, Massachusetts, USA), supplemented with 10% (v/v) heat-inactivated fetal bovine serum (FBS, Sigma-Aldrich, Saint Louis, Missouri, USA), 2 mM L-glutamine (Gibco), 100 U/ml penicillin and 100 *μ*g/ml streptomycin (Gibco) and 250 *μ*g/ml Geneticin (G418 sulfate, Gibco).

The human peripheral blood mononuclear cells (hPBMCs) isolated from healthy donors were cultured in RPMI 1640 medium (Gibco), supplemented with FBS, L-glutamine, penicillin and streptomycin as before, plus 10 mM HEPES (Gibco). All the cell cultures were grown at 37°C in a humidified atmosphere with 5% CO_2_.

### 2.2. Plasmids

The pYD1 plasmid (Invitrogen) was used for the scFv expression in yeast cells. An expression cassette, containing the GAL1 promoter and the sequence of the wild type scFv PD-L1_1, in frame with the Aga2p subunit of the a-agglutinin Aga and with the HA tag at the 3′ end, was *de novo* synthesized by the GeneArt synthesis service (Thermo Fisher Scientific) and then cloned into pYD1 using BtgZI and PmeI restriction sites. In this way, the plasmid pYD1-PD-L1_1-Aga2-HA was generated. In order to provide the highest possible display of the scFvs, the codon usage of the cassette was optimized for expression in the yeast system.

The plasmids pEU8.2 and pEU4.2 were used for expression of the mAbs in EBNA cells. These plasmids encode for the VH and VL constant regions of type 4 immunoglobulins G (IgGs), respectively, as previously described [63]. To convert the scFvs into full length IgGs, the sequences of their VH and VL were *de novo* synthesized by the GeneArt synthesis service with a human-optimized codon usage and then subcloned in pEU8.2 and pEU4.2 plasmids, respectively. VH cloning in pEU8.2 was performed using BssHII and BamHI restriction sites, whereas VL was cloned in pEU4.2 by ApaLI and AvrII sites.

### 2.3. Random Mutagenesis of HCDR3

To generate repertoires of scFvs with improved affinities for the PD-L1 protein, the HCDR3 of the PD-L1_1 scFv was randomly mutated using GeneMorph II Random Mutagenesis Kit (Agilent Technologies, Santa Clara, California, United States). 10 pg of the pYD1-PD-L1_1-Aga2-HA plasmid were amplified with the following primers: forward primer: 5′-GAAGATACTGCTGTTTACTACTGTGCTAGA-3′ and reverse primer: 5′-CAGTAACCATAGTACCTTGACCCCA-3′, and with the following reaction conditions: 30 s denaturing at 95°C, 30 s annealing at 60.3°C, 15 s extension at 72°C and 30 cycles of amplification. The template plasmid present in the amplification product was removed by enzymatic restriction with 1 *μ*l DpnI (New England Biolabs, Ipswich, Massachusetts, USA) at 37°C for 30 min. For the generation of repertoires of CDRs with different mutation frequencies, 10 pg of DNA from this mutagenesis reaction were amplified again with additional 9 sequential PCR reactions, in the same conditions as described before. After each amplification step, the PCR products were analyzed by electrophoresis on 2.5% agarose gel in 1X Tris-borate-EDTA buffer and gel-purified with Wizard SV Gel and PCR Clean-Up System (Promega, Madison, Wisconsin, United States). The DNA concentration was determined by a spectrophotometer at 260 nm. Each PCR reaction was performed in triplicate to obtain enough DNA for the subsequent steps.

### 2.4. High-Throughput Sequencing (HTS) of the Repertoires of Mutated HCDR3s

The ten different repertoires of randomly mutated CDR3s were analyzed by HTS with a MiSeq apparatus (Illumina, San Diego, California, United States). The preparation of bar-coded fragments, sequencing reactions and data analysis were performed at Center for Translational Genomics and Bioinformatics, San Raffaele Hospital, Milan, Italy. TruSeq ChIP Sample Prep Kit (Illumina) was used for the preparation of bar-coded fragments and then samples were diluted to a final concentration of 10 pM and sequenced in two runs, using 2 × 75 bp kit v3 [64].

### 2.5. Production of Full Length scFvs Containing the Mutated HCDR3s

The scFv regions located upstream and downstream the CDR3 were amplified from the plasmid pYD1-PD-L1_1-Aga2-HA using the high fidelity AccuPrime Pfx DNA polymerase (Invitrogen) and then joined to the repertoire of mutated CDR3s by assembly PCR. These upstream and downstream sequences were amplified in two separate reactions, respectively, using 10 ng of template DNA, with the following primers and reaction conditions:

Primers for the upstream sequence:
    Forward: 5′-TGAAGAGGAAAAATTGGCAGTAACC-3′ (primer 1)
    Reverse: 5′-CTAGCACAGTAGTAAACAGCAGTA-TCTTC-3′ (primer 2)
Primers for the downstream sequence:
    Forward: 5′-TGGGGTCAAGGTACTATGGTTACTG-3′ (primer 3)
    Reverse: 5′-ATAAAGTATGTGTAAAGTTGGTAA-CGGAACG-3′ (primer 4)
Amplification protocol: 15 s denaturing at 95°C, 30 s annealing at 58°C (upstream sequence) or 60°C (downstream sequence), 1 min extension at 68°C and 30 cycles of amplification

After treatment with DpnI to digest residual template DNA, the fragments were analyzed by electrophoresis on 1% agarose gel in 1X Tris-acetate-EDTA buffer and gel-purified. For assembly PCR, 10 ng of amplified upstream and downstream fragments were mixed with 10 ng of mutated CDR3s and amplification was performed with primers 1 and 4 indicated above, which were added to the reaction during the denaturing step of the 6^th^ PCR cycle. The following amplification protocol was used: 15 s denaturing at 95°C, 30 s annealing at 58°C, 1 min 40 s extension at 68°C and 30 cycles of amplification.

### 2.6. Yeast Library Generation by In Vivo Recombination

The *S*. *cerevisiae* strain EBY100 (ATCC) was streaked on a YPD plate (recipe in Supplementary [Supplementary-material supplementary-material-1]) and grown at 30°C for 36–48 h. A single colony was inoculated in 5 ml of YPD and grown overnight at 30°C in a platform shaker until the stationary phase, corresponding to approximately 5-6 optical density (OD) at 600 nm (at this wavelength, 1 OD of culture contains more or less 1 × 10^7^ yeast cells/ml [24]). 200 ml of YPD were then inoculated at 0.003 OD/ml with yeasts from the previous inoculum and grown overnight as before. When the mid-log phase was reached (which corresponded to 1.5–1.6 OD), yeast cells were harvested by centrifugation at 2000 ×g for 30 min at 4°C, washed twice with 25 ml of ice-cold filter-sterilized ultrapure Milli-Q water and once with ice-cold electroporation buffer (ICEB: 1 mM calcium chloride and 1 M sorbitol, in Milli-Q water) and centrifuged at 2000 ×g for 10 min at 4°C. Soon after, yeast cells were conditioned in 20 ml lithium acetate-dithiothreitol buffer (LAD buffer: 0.1 M lithium acetate and 10 mM dithiothreitol, in Milli-Q water) for exactly 30 min at 30°C with gentle shaking (100 rpm). Competent yeasts were collected as before and, after washing with ICEB, they were resuspended in the same buffer to reach a final volume of 1 ml and used immediately for library construction. Small aliquots (about 3.2 ×  10^8^ yeast cells) were frozen, to be used for the generation of scFv-expressing yeast clones. The ICEB and LAD buffer were prepared immediately before use and filter-sterilized.

400 *μ*l of competent cells, corresponding to approximately 1.3 × 10^9^ yeasts, were mixed with 8-9 *μ*g of gel-purified full length scFvs and 1 *μ*g of pYD1-PD-L1_1-Aga2-HA plasmid (25 : 1 molar ratio, in a final volume of not more than 40 *μ*l), which had been previously linearized with BtgzI and PmeI restriction enzymes (New England Biolabs) to allow *in vivo* recombination of scFvs. The mix was transferred to a prechilled electroporation cuvette with a 2 mm electrode gap and, after 5-minute incubation on ice, yeasts were electroporated by Gene Pulser Xcell™ (Bio-Rad, Hercules, California, USA) using the following exponential protocol: voltage 2500 V, resistance 100 ohm, capacitance 25 *μ*F. In order to reach a transformation efficiency of about 1–2 × 10^8^ transformants, two different aliquots of competent yeast cells were electroporated separately but simultaneously. After electroporation, each aliquot was immediately recovered in 8 ml of a 1 : 1 mix of YPD and 1 M sorbitol for 2 h at 30°C, in a platform shaker at 200–250 rpm. The two aliquots of electroporated cells were therefore collected at 2000 ×g for 10 min at room temperature, pooled and resuspended in 200 ml of the selective medium YNB-trp (recipe in Supplementary [Supplementary-material supplementary-material-1]). The culture was grown for 16–20 h at 30°C, in a platform shaker at 200–250 rpm. 20% (v/v) glycerol stocks were made, each containing a tenfold excess of the expected library diversity.

To determine the transformation efficiency, which also corresponded to the theoretical library diversity, serial dilutions (1 : 100, 1 : 1000, 1 : 10000 and 1 : 100000) were prepared from libraries in the selective medium and 100 *μ*l of each dilution were spread on selective plates and grown at 30°C for 36–48 h. The number of colony-forming units (CFU) was determined and the titer was calculated as the average of the counts obtained for each plate.

### 2.7. Flow Cytometry Binding Assays of Yeast Cells to rhPD-L1 Protein

Yeast cultures were induced overnight for 18 h in the induction medium (recipe in Supplementary [Supplementary-material supplementary-material-1]) at 21°C at 0.5 OD/ml, in a shaking incubator at 200–250 rpm, in order to allow the expression of scFvs on their surface. 1 × 10^6^ induced yeast cells were washed once with 200 *μ*l phosphate buffered saline 1X (PBS, Sigma-Aldrich) 0.5% (w/v) albumin from bovine serum (BSA, Sigma-Aldrich), in a round-bottom 96-well plate, and centrifuged at 2000 ×g for 3 min at room temperature. Cells were incubated with recombinant human PD-L1, Fc-tagged (rhPD-L1-Fc, R&D Systems, Minneapolis, Minnesota, USA), and diluted in 100 *μ*l washing buffer, for 30 min at room temperature in a platform shaker with gentle agitation. To perform the binding curve of the wild type yeast, tenfold serial dilutions of rhPD-L1-Fc were used (from 10 pM to 10 nM, plus an additional saturation concentration of 30 nM), whereas 10 pM only was used to screen the selected yeast clones. A sample incubated with buffer only was used as blank. After binding of PD-L1, cells were incubated for 5 min on ice and then washed once as before. This step and the following ones were performed at 4°C to prevent dissociation of PD-L1 from scFv-expressing yeasts. For labeling of yeast-PD-L1 complexes, 1 *μ*l of Alexa Fluor 488-conjugated anti-HA antibody (BioLegend, San Diego, California, USA) and 1 : 2000 APC-conjugated anti-human IgG, Fc*γ* specific antibody (Jackson ImmunoResearch, West Grove, Pennsylvania, USA) were used in 100 *μ*l washing buffer. After 30 min incubation in agitation in the dark, cells were washed twice and then resuspended in 200 *μ*l PBS 1X and fluorescence was detected by the CytoFLEX flow cytometer (Beckman Coulter, Brea, California, United States). Data analysis was performed by CytExpert software (Beckman Coulter). An unstained sample was used to fix fluorescence cutoff.

### 2.8. Yeast Library Screening by Cell Sorting

For the first selection round, a tenfold excess of the library diversity was induced as before. 1 × 10^8^ total induced yeast cells, split into 10 different aliquots of 1 × 10^7^ cells each, were incubated with 30 or 10 nM rhPD-L1-Fc, in 200 *μ*l washing buffer in 2 ml tubes. After one washing with 1 ml PBS 1X 0.5% BSA, the two fluorescent antibodies used for labeling of yeast-protein complexes were added at the same concentrations indicated above, in a final volume of 200 *μ*l washing buffer and incubated for 30 min in the dark. Cells were then washed twice and the pellet was kept on ice in the dark until sorting. Just before sorting, each aliquot of labeled cells was resuspended in 1.5 ml PBS 1X 0.5% BSA; sorting was performed with FACSAria IIIu (BD Biosciences, Franklin Lakes, New Jersey, United States) at the flow rate of 5000 evts/sec and data analysis was performed by FACSDiva 6.1.3 software (BD Biosciences). Sorted cells were recovered in 3-4 ml of the selective medium, grown for 24 h until saturation and then induced at a tenfold excess of their diversity for the next selection step.

For the subsequent rounds of selection, 1 × 10^7^ total cells were stained. For library 3, two more sortings were performed, using 30 nM and 10 pM rhPD-L1-Fc, respectively. For library 10, one more sorting was performed at 10 nM. Cells were resuspended in 3 ml PBS 1X 0.5% BSA, sorted with a lower flow rate (1500 evts/sec) and recovered as described before.

For sortings, a diagonal gate was chosen which included the APC-brightest yeast-PD-L1 complexes among the double-positive cells.

### 2.9. Evaluation of Diversity of Sorted Yeast Populations

The procedure for the extraction of DNA from sorted yeast cultures was adapted from the QIAprep Spin Miniprep Kit Protocol (Qiagen, Hilden, Germany), with some modifications. In brief, 60 OD of fresh overnight yeast cell culture were harvested at 2000 ×g for 10 min at 4°C and resuspended in 750 *μ*l buffer P1. 500 *μ*l of acid-washed glass beads (Sigma-Aldrich) were added to the cell suspension and the yeast cell wall was mechanically destroyed by the tissue lyser (Qiagen) for 2 min at 30 Hz. Chemical lysis and subsequent neutralization were performed in 750 *μ*l of buffers P2 and N3, respectively. Lysate clarification and DNA binding to the column were performed according to the QIAprep Spin Miniprep Kit handbook. After washing with 500 *μ*l buffer PB and 750 *μ*l buffer PE, DNA was eluted in 30 *μ*l UltraPure DNase/RNase-Free Distilled Water (Gibco) and its concentration was determined by a spectrophotometer at 260 nm.

As the quality of DNA from yeast cultures is not suitable for sequencing because of a high contamination from polysaccharides of the yeast cell wall, 200 ng of this plasmid DNA were used to transform 50 *μ*l of high-efficiency chemically competent *E*. *coli* DH10B cells (Invitrogen), according to the manufacturer's recommendations, and all the transformed cells were spread on a single plate. 24 random bacterial colonies were picked, plasmid DNA was extracted with QIAprep Spin Miniprep Kit and then the CDR3 of each clone was sequenced with the forward primer 5′-CTCCAGGTAAAGGTTTGGAATGGG-3′. 30–40 ng of this DNA were retransformed into 3.2 × 10^8^ yeast competent cells, in order to generate the individual yeast clones expressing the sequenced scFvs. Cells were electroporated with MicroPulser (Bio-Rad) using the preset protocol Sc2 and then recovered as described for the generation of the libraries and plated on a single YNB-trp plate.

### 2.10. mAb Production and Purification

15 *μ*g of the plasmid pEU8.2 and 15 *μ*g of the plasmid pEU4.2 were cotransfected in EBNA 293 cells grown in 150 mm tissue culture dishes at 70–80% confluency. Lipofectamine reagent 2000 (Invitrogen) was used for transfection according to the manufacturer's protocol. 6 h after transfection, the culture medium was replaced by the serum-free medium CD CHO (Gibco), supplemented with 2 mM L-glutamine (Gibco), 100 U/ml penicillin and 100 *μ*g/ml streptomycin (Gibco), 250 *μ*g/ml Geneticin (G418 sulfate, Gibco) and 2.5 *μ*g/ml amphotericin B (Gibco). After 7–10 days, media containing the antibodies were collected and clarified at 2000 rpm for 30′ at 4°C.

The mAbs were purified from the culture medium by protein A affinity chromatography, using Protein A HP SpinTrap (GE Healthcare, Chicago, Illinois, USA). Protein A resin was removed from the column, added to the culture medium (about 20 ml for each antibody) and then incubated for 3 h at room temperature with low rotation. After centrifugation at 100 ×*g* for 30 sec, the medium was discarded and the resin, bound to the mAb, was resuspended in 600 *μ*l of binding buffer (GE Healthcare) and reloaded onto the column. The subsequent washing and elution steps were performed as indicated by the supplier.

The antibody concentration in the eluted fractions was determined using Pierce BCA Protein Assay Kit (Thermo Fisher Scientific). The molecular weight of the antibodies and the purity of the preparations were checked by sodium dodecyl sulfate-polyacrylamide gel electrophoresis (SDS-PAGE) in reducing and not-reducing conditions, followed by Coomassie staining.

### 2.11. Kinetic Analyses by Surface Plasmon Resonance (SPR)

SPR analyses were performed at 25°C in a Biacore X100 instrument (GE Healthcare), equipped with CM5 sensor chips (GE Healthcare). The HBS-EP buffer (10 mM HEPES, 0.15 M NaCl, 3 mM EDTA and 0.05% surfactant P20 at pH 7.4) was used as the running buffer (GE Healthcare). Kinetic and equilibrium dissociation constants were measured for mono- and bivalent complexes by two different experimental strategies.

To investigate the monovalent binding properties of soluble rhPD-L1-his (R&D Systems) to anti-PD-L1 IgGs, a capturing method was chosen. To this purpose, protein A from *Staphylococcus aureus* (Thermo Fisher Scientific) was immobilized onto the surface of a CM5 sensor chip using standard amine coupling chemistry. In brief, the sensor surface was firstly activated by injecting a 1 : 1 (v/v) mixture of 0.4 M 1-ethyl-3-(3-dimethyl aminopropyl)carbodiimide hydrochloride (EDC) and 0.1 M *N*-hydroxysuccinimide (NHS) at a flow rate of 10 *μ*l/min for 7 min. Protein A (50 *μ*g/ml) dissolved in 10 mM sodium acetate buffer, pH 4.75, was then injected at a flow rate of 5 *μ*l/min, followed by the injection of 1 M ethanolamine hydrochloride, pH 8.5, for 7 min at a flow rate of 10 *μ*l/min. Typically, 2000 response units (RU) of protein A were immobilized. The reference flow cell was inactivated by amine coupling chemistry as already described, omitting the protein A injection. IgGs (2 *μ*g/ml in the HBS-EP running buffer) were injected onto the chip at a flow rate of 5 *μ*l/min, giving typically capture levels of about 200 RU. Binding of rh-PD-L1-his to the captured IgGs was analyzed by injecting twofold serial dilutions in the HBS-EP running buffer as follows: 500–15.62 nM (wild type mAbs) and 250–7.81 nM (mutated mAbs). rhPD-L1-his was injected at a flow rate of 30 *μ*l/min and the association phase was recorded for 180 s contact time. Dissociation of rh-PD-L1-his was observed for 300 s. The sensor surface was regenerated by one 30 s injection of 25 mM NaOH at a flow rate of 10 *μ*l/min. A blank curve in which the running buffer only was passed over the captured antibodies was subtracted from each sensorgram.

Association rate constants (*k*_a_) and dissociation rate constants (*k*_d_) were determined by fitting the curves to the 1 : 1 Langmuir binding model, using the Biacore X100 Evaluation Software (version 2.0.1). Values of *χ*^2^ for fittings were ≤1, indicating good fits. The equilibrium dissociation constants (*K*_D_) were calculated according to the relationship *K*_D_ = *k*_d_/*k*_a_. Steady-state analyses were also carried out to calculate the equilibrium dissociation constants (*K*_D1_) of wild type and mutated mAbs by nonlinear curve fitting of 1 : 1 Langmuir binding isotherms, plotting equilibrium response toward analyte concentrations. The equilibrium dissociation constants (*K*_D1_) were calculated from the concentration corresponding to half-maximal saturation. Kinetic parameters and standard deviations were obtained from at least four independent analyses using different biosensors, sample preparations, ligand densities and analyte concentration gradients on the flow cell surfaces.

A second set of SPR experiments was carried out to determine the apparent affinity constants (*K*_D2_) for the bivalent complexes. rhPD-L1-Fc (300 RU; disulfide-linked homodimer) was immobilized by standard amine coupling chemistry onto the CM5 sensor chip, and the reference flow cell was blocked as described above. Binding of IgGs to the immobilized rhPD-L1-Fc was analyzed by injecting twofold serial dilutions in the HBS-EP running buffer as follows: 12.5–0.39 nM (wild type mAbs) and 6.25–0.39 nM (mutated mAbs). mAbs were injected at a flow rate of 30 *μ*l/min and the association phase was recorded for 180 s contact time. Dissociation of mAbs was observed for 600 s. The sensor surface was regenerated by one 30 s injection of 5 mM NaOH at a flow rate of 10 *μ*l/min. A blank curve was used, as before.

### 2.12. Binding Assays of mAbs to hPBMCs

hPBMCs were isolated from blood of healthy donors by using the Greiner Leucosep® tube (Sigma-Aldrich) following the manufacturer's instructions and frozen in a solution containing 90% FBS (Sigma-Aldrich) and 10% dimethyl sulfoxide (DMSO, Sigma-Aldrich) until use.

hPBMCs were thawed in RPMI 1640 medium, supplemented with 2 mM L-glutamine, 1% (v/v) of CTL wash supplement (Cellular Technology Limited, Shaker Heights, Ohio, USA) and 100 U/ml Benzonase (Merck Millipore, Burlington, Massachusetts, United States), and collected at 1200 rpm for 10 min. They were resuspended in the complete RPMI medium (as described in Section 2.1) and plated at about 1 × 10^7^ cells/well in a 12-well plate. After resting for 16 h at 37°C, hPBMCs were counted by using the Muse® Cell Analyzer (Merck Millipore), resuspended at the concentration of 1 × 10^6^ viable cells/ml in the complete medium in a 12-well plate and activated with Dynabeads Human T-Activator CD3/CD28 (Gibco), 25 *μ*l beads/1 × 10^6^ viable cells. 24 h after activation, the cells were collected and plated at 4 × 10^5^ cells/well in 100 *μ*l PBS in a 96-well plate with round bottom. After one wash in PBS, they were incubated with 50 *μ*l LIVE/DEAD™ Fixable Violet Dead Cell Stain (Invitrogen) for 30 min at +4°C and washed once again. The wild type and affinity matured mAbs, diluted in 100 *μ*l PBS at concentrations ranging from 9.6 pM to 6 nM, were added to the cells and incubated for 1 h 30 min at room temperature in the dark with gentle shaking. The cells were washed twice with PBS and then incubated with 5 *μ*l of PE-conjugated anti-human CD2 antibody (BD Biosciences) and 1 : 2000 APC-conjugated anti-human IgG, Fc*γ* specific antibody (Jackson ImmunoResearch), diluted in 100 *μ*l PBS 1X 1% (v/v) FBS (FACS buffer). After 45 min incubation as before, two washes with the FACS buffer were performed and cells were then resuspended in 150 *μ*l PBS for the acquisition by the CytoFLEX flow cytometer (Beckman Coulter).

### 2.13. Effects of Affinity-Matured mAbs on the Secretion of Cytokines by Stimulated hPBMCs

hPBMCs (1 × 10^6^ cells) were cultured and stimulated with 2.5 *μ*g/mL phytohemagglutinin-L (PHA-L, Sigma-Aldrich) or 50 ng/ml staphylococcal enterotoxin B (SEB, ELISA assay us Sigma-Aldrich) for 18, 42, and 66 hours, in the absence or the presence of anti‐PD-L1_1 mAb (20 *μ*g/mL) or its derived variant 10_3 obtained by affinity maturation. An unrelated isotype antibody was used as the negative control. The levels of interleukin 2 (IL-2) and interferon-*γ* (IFN-*γ*) in cell culture supernatants collected at the timepoints indicated above were measured by ELISA assay using DuoSet ELISA (R&D Systems) according to the manufacturer's recommendations. Each sample was analyzed in triplicate. Analyses were performed by using samples of hPBMCs obtained from at least two different donors. Error bars were calculated on the basis of the results obtained by at least two independent experiments. Differences between groups were assessed by Student's *t*-test. Statistical significance was defined as *p* ≤ 0.001.

## 3. Results

### 3.1. Multistep Random Mutagenesis of an Anti-PD-L1 scFv and High-Throughput Sequencing of Different Repertoires of Mutants

An scFv specific for PD-L1 protein, named “PD-L1_1,” was previously identified from a phagemid library [62]. To improve the affinity of this scFv (hereafter called wild type) for PD-L1, we generated repertoires of mutants by CDR-targeted random mutagenesis, just restricted to the CDR3 (33 bp in length) of its VH. To obtain a repertoire of sequences containing the maximum possible diversity of mutants and a very low frequency of the wild type sequence, we amplified the CDR3 by 10 sequential cycles of error-prone random mutagenesis and carried out a systematic analysis by high-throughput sequencing on the pools of CDR3s obtained at each mutagenesis step.

The ten pools of PCR fragments were differently bar-coded and sequenced in two runs in a MiSeq apparatus. To be sure of the correct sequence of each fragment, both forward and reverse strands of each molecule were sequenced and the number of reads obtained (i.e., the number of sequenced molecules) was in comparable orders of magnitude for all samples (between 5 × 10^5^ and 2.2 × 10^6^).

To evaluate the quality and complexity of the CDR pools, we evaluated several parameters by means of bioinformatics analysis, i.e., the frequency of wild type CDR3 sequences, the percentage of stop codon-containing reads and the abundance of single and multiple mutations.

**Figure 1:**
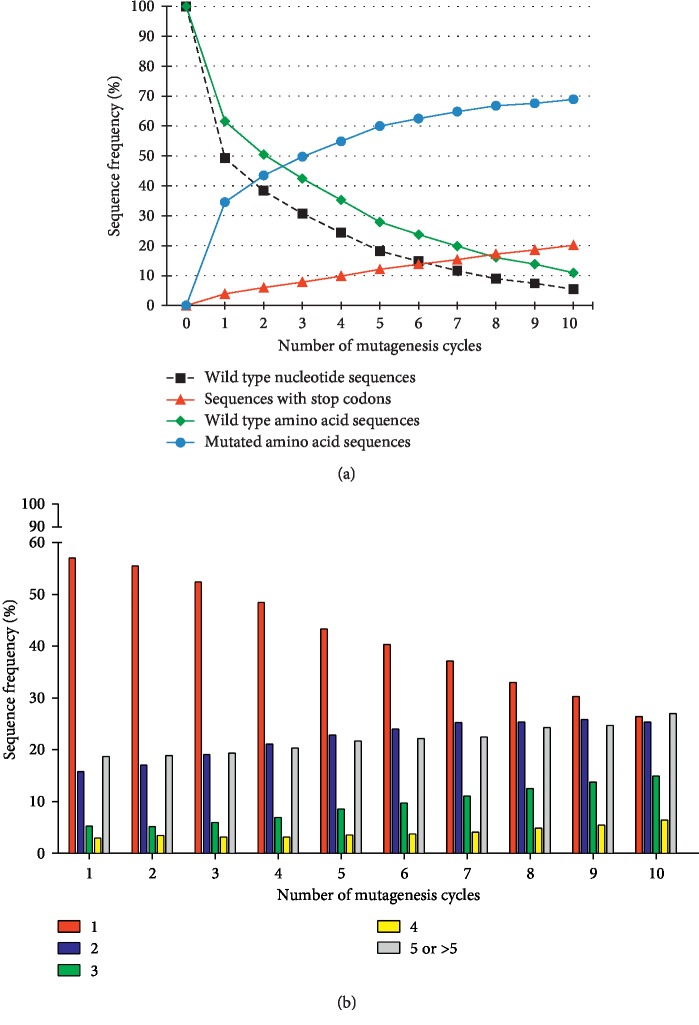
High-throughput sequencing analysis of the CDR3 fragments obtained by random mutagenesis. (a) The chart shows the relative representation of wild type and mutated CDR3 sequences in the indicated cycles of mutagenesis. To estimate the efficiency of mutagenesis, the fraction of fragments showing the nucleotide wild type sequence was evaluated (dotted black line). The amino acid sequences of all the CDR3 fragments were analyzed too, to distinguish between CDR3s showing the wild type sequence (green line-rhomboids) and those containing missense substitutions (blue line-circles) or stop codon triplets (red line-triangles). (b) The histogram represents the distribution of single and multiple mutations in the pools of CDR3s throughout the mutagenesis. The translated sequences were grouped according to the number of amino acid substitutions (indicated in the legend) and are shown as percentage of the mutated sequences.

To test the efficiency of the multistep mutagenesis, we evaluated the frequency of wild type nucleotide sequences, which showed an inverse correlation with the number of mutagenesis rounds (Figure 1(a)), following a non‐linear trend throughout the mutagenesis. In particular, after a fast drop of 50%, the magnitude of this decrease became progressively lower, reaching 95% reduction of wild type after 10 rounds.

We carried out the same analysis after translating all the reads into their corresponding amino acid sequence. As shown in Figure 1(a), the reduction trend of the CDR3s with an amino acid wild type sequence was the same as that one observed for the nucleotide sequences; however, in each round, more translated wild type CDR3s than nucleotide ones were found (from 5% to 12% more) because of the presence of sequences containing silent mutations.

The fraction of reads containing stop codons was also evaluated as a measure of the number of truncated scFvs that were likely to be lost during selection because not expressed or unable to bind to the antigen. As expected, the higher the number of point mutations accumulated in the sequences, the higher the chance to introduce stop codon triplets. In fact, these sequences constantly increased at each mutagenesis step with a linear trend.

The remaining fraction of reads of each round was represented by sequences carrying missense mutations. The first round alone was able to increase their percentage up to 35%, while from round 2 onwards, these mutated sequences increased very slowly, up to a peak of 70% at round 10.

In addition, we also studied the abundance of single, double, triple, or multiple mutations. As shown in Figure 1(b), reads containing 1 substitution were the most abundant mutated sequences in almost all rounds; they represented more than half of the mutated reads at round 1 (57%) and decreased up to round 10, where their fraction was about twofold lower than that in the first round. On the contrary, the percentage of reads with 2 mutations slowly increased throughout the mutagenesis, reaching the same level as those containing 1 mutation at round 10. CDR fragments carrying 3 mutations increased too, while those containing 4 mutations slightly increased only in the last rounds. Sequences that accumulated 5 or more mutations (up to 25) were found, but they were less frequent; in fact, each of these subgroups corresponded to a variable, but low fraction of the total reads (ranging from 0.1% to 3%), depending on the mutagenesis round. However, if considered all together, they ranged from 18% of mutated reads at round 1 to 27% at round 10. We found up to 31 mutations in some sequences, but these cases were indeed very rare, representing only 0.001–0.006% of the mutated reads in some cycles.

### 3.2. Generation of scFv-Displaying Yeast Libraries and Cell Sorting-Based Selection of High Affinity Anti-PD-L1 scFvs

To select the scFv variants with improved affinity for PD-L1, we used a yeast display platform, in which the antibody fragments were expressed on the *S*. *cerevisiae* surface (as fusion proteins with the cell wall a-agglutinin Aga) and then screened for their binding properties to the target antigen. Considering our aim to improve the affinity maturation platform and basing on the overall distribution of mutations, we generated two yeast libraries using the mutated CDR3 fragments coming from two different cycles, number 3 and 10, respectively (as described in Materials and Methods), and then compared the results of the two selections. In round 3, the percentage of mutated sequences started to exceed that of the wild type and the majority of them harbored single point mutations; on the contrary, round 10 corresponded to the condition in which the fraction of wild type sequences was the lowest of all rounds and sequences carrying single and double mutations were present at the same level (Figures 1(a) and 1(b)). Considering that the maximal theoretical diversity achievable in yeast libraries is 10^9^, high and comparable titers (i.e., the number of scFv clones) were obtained for both libraries: about 2 × 10^8^ clones for the library generated with fragments coming from cycle 3 (hereafter library 3) and approximately 1.7 × 10^8^ clones for the library obtained using CDR3s from cycle 10 (hereafter library 10).

For the isolation of high affinity scFvs from the two libraries, scFv-expressing yeast cells were incubated with appropriate PD-L1 concentrations, and the top binders were separated and enriched by various rounds of cell sorting, increasing the selection stringency; in particular, at each round, PD-L1 concentrations were reduced and/or a more selective sorting gate was chosen.

We used a recombinant human chimeric version of PD-L1 for the selections of the libraries; this protein was a soluble homodimer of two chimeric subunits, each formed by the human PD-L1 extracellular domain, linked to the Fc region of human IgG1 (rhPD-L1-Fc). The Fc fragment only worked as tag for the detection of the protein by flow cytometry (see Materials and Methods). Although PD-L1 is a monomer in its physiological context, that is, when expressed on the plasma membrane of human cells, and although monomeric PD-L1 is commercially available, we preferred to use the same PD-L1 format as that used for the *de novo* isolation of the wild type PD-L1 specific scFv in a phage display system [62].

**Figure 2:**
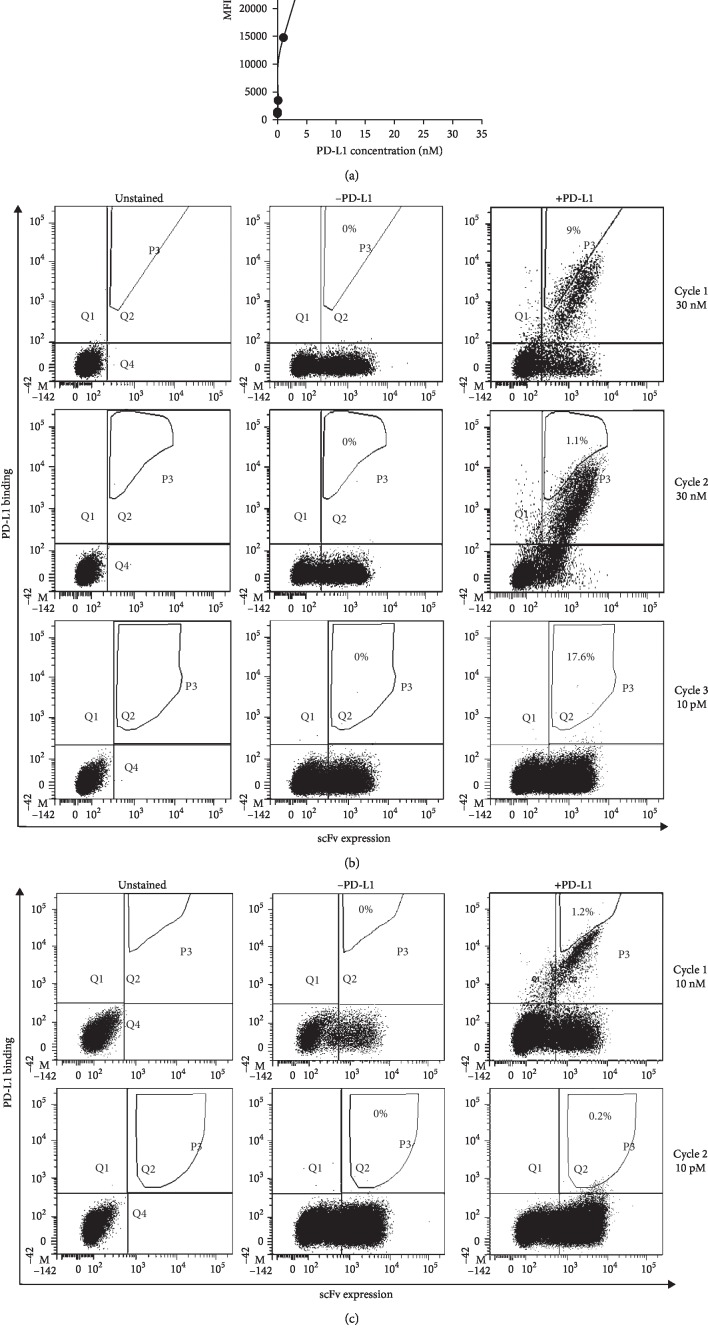
Strategy for the isolation of high affinity scFvs from the yeast libraries. (a) The graph reports the binding curve of the wild type yeast to recombinant human PD-L1, a dimer of two identical molecules, each containing the PD-L1 extracellular region linked to the Fc tag (rhPD-L1-Fc). The yeast clone expressing the wild type scFv was incubated with increasing rhPD-L1-Fc concentrations and analyzed by flow cytometry. The mean fluorescence intensity of the anti-Fc antibody, used to detect the yeast-PD-L1 complexes, was plotted as a function of rhPD-L1-Fc concentration. (b and c) The panels represent different cell sorting strategies used for the selection of high affinity clones from library 3 (b) and library 10 (c). Yeast cells were stained with two fluorescent antibodies, detecting the scFvs and the rhPD-L1-Fc antigen, respectively. At each sorting cycle, unstained samples (left columns) were used to determine fluorescence thresholds, while samples stained in absence of the target antigen (middle columns) were used to exclude any non‐specific signal from the sorting gate. The sorted yeasts are shown in the polygonal gate (right plots), and they are expressed as percentage of PD-L1-binding cells (present in the upper right quadrant). rhPD-L1-Fc concentrations used for each selection cycle are indicated on the right.

For library 3, we performed three rounds of selection; in order to cover the diversity of the mutants present in the starting library, in the first round of selection we used a number of yeasts comparable to the titer of the library (1 × 10^8^ cells) and incubated them with a saturating concentration of PD-L1 (30 nM), thus avoiding that antigen concentration would be limiting for the rare but high affinity yeast clones. PD-L1 concentrations were properly determined by performing a binding curve of dimeric rhPD-L1-Fc to the yeast clone expressing the wild type scFv (Figure 2(a)). About 9% of the PD-L1 binding cells was selected and separated through a diagonal sorting gate (Figure 2(b), upper panels), thus including the yeasts with the highest fluorescence intensity for the antigen binding, independently from their scFv expression level. The recovered cells (about 3 × 10^5^) were then amplified and sorted again. For the second selection round, 1 × 10^7^ cells amplified from the previous step (an excess compared to the theoretical diversity of the sorted ones) were incubated with the same PD-L1 concentration as before but selected by a more stringent sorting gate, including only 1.1% of the yeast-PD-L1 complexes (Figure 2(b), middle panels). The 2 × 10^4^ sorted yeasts were amplified and, before going on with additional rounds, we checked some of them (24 clones) by Sanger sequencing. As only 17.4% of the sorted clones carried a mutation in the CDR3 amino acid sequence (data not shown), a great loss of mutants occurred during these selection steps considering that the fraction of mutated CDR3s produced by the mutagenesis was 50% at cycle 3. For this reason, in an attempt to counterselect the wild type yeasts still present at a high percentage, we performed a third selection round with very stringent conditions, in which the wild type yeast showed a very low PD-L1 binding level (10 pM PD-L1) (Figure 2(a)). In this condition, only 0.05% of the scFv-expressing cells from the second sorting was able to bind the antigen and, although not clearly visible, the sorting gate included 17.6% of these antigen-binding yeasts (Figure 2(b), lower panels). By this strategy, we sorted only 75 total yeasts, from 1 × 10^7^ starting cells.

We adopted a different sorting scheme for the selection of mutated scFvs from library 10. In order to quicken the overall procedure, we performed only two sorting rounds with a higher selection stringency (Figure 2(c)). In the first round, we incubated 1 × 10^8^ cells of the library with 10 nM PD-L1 (which resulted in the same binding saturation level as 30 nM, as shown in Figure 2(a)); however, in order to avoid the selection of the wild type yeast since the beginning of the sorting, we restricted the sorting gate to only 1.2% of the PD-L1 binding cells, leading to the recovery of 5 × 10^4^ yeasts. The second and last round was performed in the same conditions as the third round of library 3; therefore, we incubated 1 × 10^7^ cells amplified from the first sorting with 10 pM PD-L1, obtaining a very low percentage of binding yeasts. We selected 0.2% of the yeast-PD-L1 complexes, which resulted in the recovery of about 900 yeast cells.

### 3.3. Sequencing of Random Clones from the Selected Yeast Populations and Validation of Their Binding Improvement

To evaluate the effectiveness of the selections in reducing the wild type clones and enriching the mutant ones, we extracted the plasmid DNA from the pool of the sorted cells and sequenced the scFvs of 24 randomly chosen clones from each sorted library. Among the clones sequenced from sorted library 3, we found 75% mutated clones (18 of 24 sequences), containing single or double mutations, while only 25% of them carried the wild type CDR3 (Table 1). Interestingly, the mutations always affected the same CDR3 positions; in particular, in the first position, a Thr-to-Ser substitution was always found, except for a single clone with a different mutation. In the sequences with two mutations, this Ser was always coupled with another substitution at the tenth amino acid Asp (replaced by Asn in two clones, Gly in seven clones, Ala in two clones and Tyr in one clone). A single clone carrying a substitution only at the tenth position was also found.

Among clones sequenced from sorted library 10, no wild type clones were found, in fact all of them carried one, two or even three mutations (8.3%, 75% and 16.6% of the total clones, respectively), which affected the first, the tenth and, less frequently, the ninth CDR3 amino acids (Table 2). In the double mutants, which represented the majority of these clones, a mutation at the tenth position (Asp replaced by a Gly or an Asn) was usually combined with the same substitution at the first position (a Thr-to-Ser substitution), except for three clones, in which the other mutation affected the ninth amino acid instead of the first one (a Tyr-to-Phe substitution). The mutants with the combined mutations Ser + Gly and Ser + Asn were the most enriched ones (9 and 6 copies, respectively) and they were also found in sorted library 3. In the triple mutant clones, the Ser + Gly combination was coupled with a Phe in the ninth position or, only in one clone, with an Asn at the eleventh amino acid. In the remaining clones, a single amino acid substitution was found at the ninth position, which corresponded to the same mutation found in some double or triple mutants.

**Figure 3:**
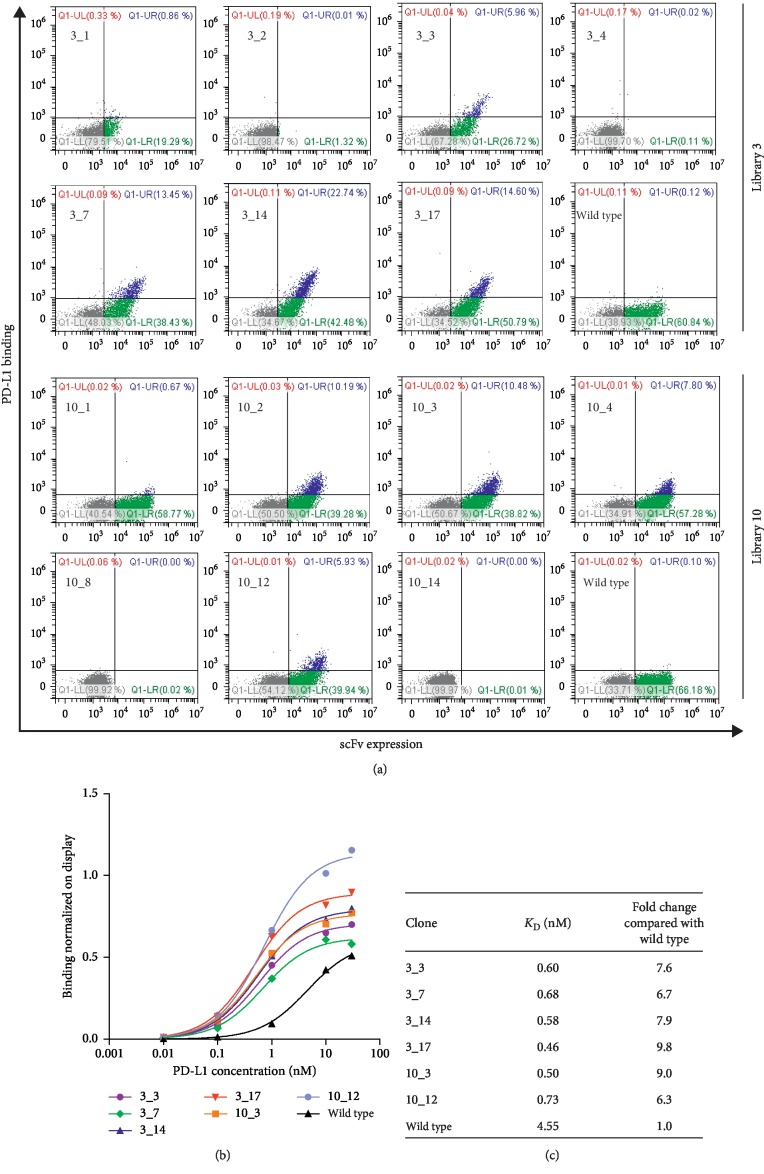
Validation of affinity improvement of the selected yeast clones. (a) The dot plots show the binding of all different mutated clones to dimeric rhPD-L1-Fc, in comparison with the wild type clone. As preliminary affinity screening, only the PD-L1 concentration used in the last sorting (10 pM) was tested. For most clones (whose name is indicated in the plots), a population of yeasts bound to rhPD-L1-Fc appeared at this concentration (blue population in the upper right quadrant), which was almost undetectable in the wild type clone. The fluorescence thresholds were fixed using an unstained control (not shown). (b) The chart represents the titration curves of the yeast-scFv clones to dimeric rhPD-L1-Fc, performed by flow cytometry. The mean fluorescence intensity of PD-L1 binding was normalized on the scFv expression, and this ratio was plotted against rhPD-L1-Fc concentration. For the two clones in common to both libraries, only the copy from library 3 is shown (clones 3_3 and 3_14). (c) The table reports the half-saturating antigen concentrations (*K*_D_) of the clones and their relative binding improvement, expressed as fold change compared with the wild type yeast.

To confirm that all the different mutated scFvs identified by sequencing analysis really had an improved affinity for PD-L1, the yeast clones expressing each of these scFvs were analyzed for their binding to dimeric rhPD-L1-Fc (the same protein as that used for the selection) in comparison with the wild type yeast, at the same antigen concentration used for the last sorting (10 pM). Among seven different clones identified in sorted library 3, four of them showed an improved binding (clones 3_3, 3_7, 3_14 and 3_17 in Figure 3(a)). The scFvs expressed by these clones corresponded to all the double mutants. Only one clone showed a very weak binding and a very low scFv expression (clone 3_1), while the remaining clones (number 3_2 and 3_4) did not express their respective scFvs at all.

A similar result was obtained for the clones selected from library 10, which were tested in an independent experiment (Figure 3(a)); in fact, except for two clones that did not display their scFv (clones 10_8 and 10_14), four out of the seven different clones bound to PD-L1 better than the wild type one (clones 10_2, 10_3, 10_4 and 10_12) and, interestingly, two of them (10_2 and 10_4) were selected also from library 3 (3_14 and 3_3, respectively). Only one clone (10_1) bound very weakly to PD-L1, despite its good expression level.

To estimate the magnitude of their binding improvement, we performed binding curves of the clones to dimeric rhPD-L1-Fc. In this determination of the apparent affinity constant for yeast-scFv clones, we considered it appropriate to use the same format of the antigen as that used for the yeast selection, to confirm that the experimental setting used for selection really worked.

To avoid that the scFv expression level could influence the result, the mean fluorescence intensity related to the binding level of the yeast-PD-L1 complexes was normalized to the mean fluorescence intensity of their scFv expression, and this ratio was plotted as a function of the antigen concentration (Figure 3(b)). The apparent *K*_D_ values (corresponding to the half-saturating antigen concentration) of all the tested clones were in the low nanomolar range (<1 nM; Figure 3(c)), and they were between 6.3-fold and 9.8-fold higher than that of the wild type clone, thus confirming that these mutated scFvs really bound to dimeric rhPD-L1-Fc with a higher affinity when expressed on the yeast surface.

### 3.4. Surface Plasmon Resonance Studies of the Affinity of the Full Length Antibodies Generated from the Selected scFvs

To assess whether the mutated scFvs retained improved affinity for PD-L1 protein also in the format of full length antibodies, their sequences were converted into full length type 4 IgGs and produced in a mammalian cell system, in order to perform surface plasmon resonance studies.

We firstly tested binding properties of 1 : 1 monovalent complexes between PD-L1 and anti-PD-L1 IgGs. To this purpose, we chose a capture method of IgGs by using the protein A immobilized chip, whereas monovalent rhPD-L1-his was used as the analyte, in place of dimeric rhPD-L1-Fc previously used for yeast selection and apparent *K*_D_ determination of yeast clones. Since IgGs are bivalent molecules (they have two antigen binding sites), the monomeric format of the analyte was necessary to study the affinity of a single antibody binding site, by evaluating the formation of monovalent complexes. In addition, the absence of Fc tag avoided the capture of recombinant PD-L1 onto the protein A immobilized chip, unlike what happened for IgGs.

**Figure 4:**
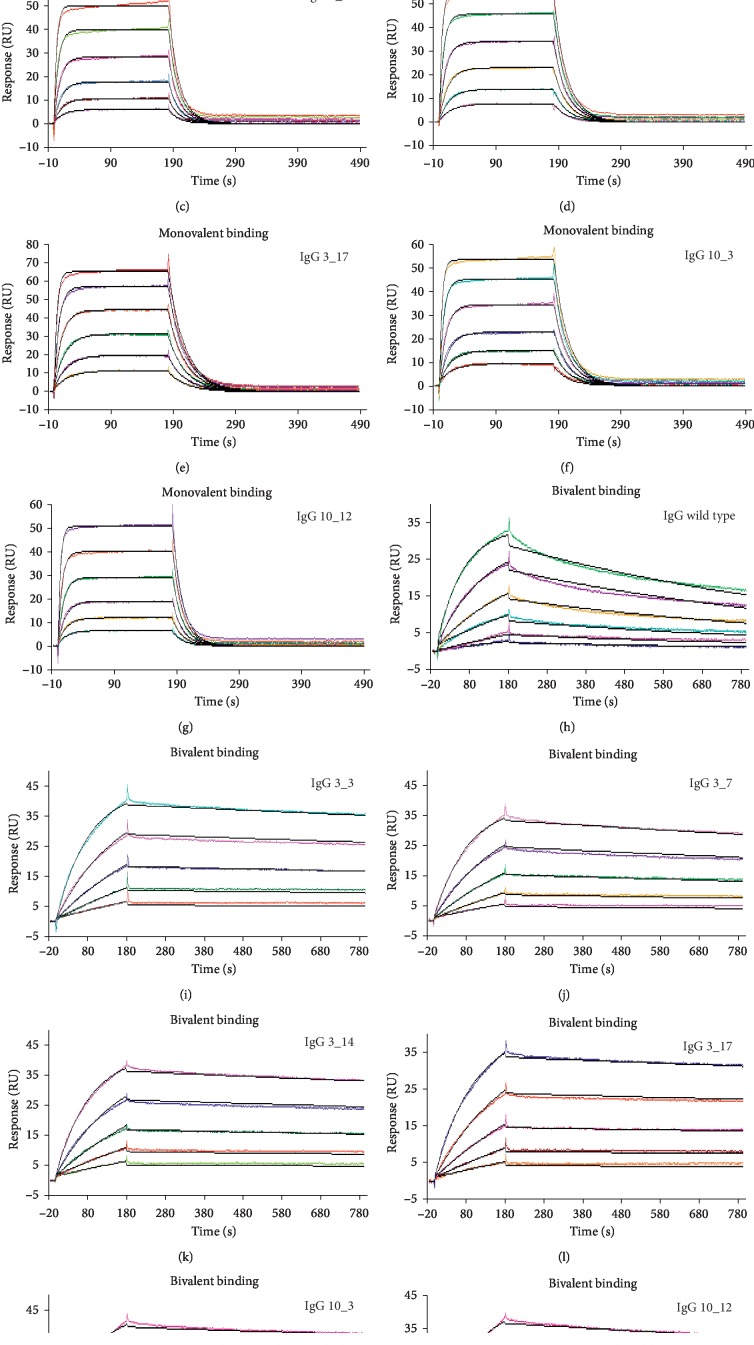
SPR binding analyses of mono- and bivalent complexes between rhPD-L1 and anti-PD-L1 IgGs. (a–g) Monovalent complexes: sensorgrams of multicycle kinetics, representative of monomeric rhPD-L1-his monovalent binding to anti-PD-L1 IgGs (colored curves), are superimposed on the calculated curves (black), fitted to the 1 : 1 Langmuir binding model. IgGs were captured (about 200 RU) onto the CM5-protein A chip, and binding of monomeric rhPD-L1-his to IgGs was analyzed by twofold serial dilution injections of rhPD-L1-his as follows: 500–15.62 nM (a) and 250–7.81 nM (b–g). (h–n) Bivalent complexes: sensorgrams of multicycle kinetics, representative of anti-PD-L1 IgGs binding to dimeric rhPD-L1-Fc (colored curves), are superimposed on the calculated curves (black), fitted to the 1 : 1 Langmuir binding model. Dimeric rhPD-L1-Fc was immobilized onto the CM5 chip by amine coupling chemistry, and binding of IgGs to rhPD-L1-Fc was analyzed by twofold serial dilution injections of IgGs as follows: 12.5–0.39 nM (h) and 6.25–0.39 nM (i–n).

The wild type IgG showed an equilibrium dissociation constant of 314 nM (*K*_D_; Table 3), indicating a weak interaction characterized by a very fast binding kinetic (Figure 4(a)) in both the association (*k*_a_ = 5.12 × 10^5^ 1/Ms) and dissociation phases (*k*_d_ = 1.61 × 10^−1^ 1/s). Binding reached equilibrium rapidly in a few seconds with a complete runoff of the analyte within 30 s after the end of PD-L1 injection. However, binding analyses of mutated IgGs showed lower equilibrium dissociation constants (*K*_D_; Table 3) compared to wild type IgG, with a decrease ranging between 6.4-fold (mAb 3_17) and 3.7-fold (mAb 3_7). Such improvement of binding properties was mainly related to the lower dissociation rate constants (*k*_d_) in comparison with that measured for wild type IgG (Table 3), indicating a more stable binding, whereas association rate constants (*k*_a_; Table 3) were almost similar to that of the wild type IgG, with a maximum increase of 1.6-fold observed for mAb 10_3. Steady-state analyses were also carried out to measure the equilibrium dissociation constants (*K*_D1_; Table 3), by plotting equilibrium response toward analyte (PD-L1) concentrations. *K*_D1_ values (corresponding to the half-saturating PD-L1 concentration) were found to be in good agreement with *K*_D_ values from kinetic analyses (Table 3), thus confirming kinetic parameters.

On the contrary, when kinetic binding properties of bivalent complexes were studied, SPR analyses highlighted stronger interactions as a consequence of avidity, as shown in Figures 4(h)–4(n). To test bivalent binding, we immobilized dimeric rhPD-L1-Fc by amine coupling chemistry at low density (300 RU) to allow the bivalent formation of 1 : 1 complexes when IgGs flowed over the chip as analytes. Such experimental conditions aimed to mimic the physiological binding conditions of IgGs on activated cells endowed with very high PD-L1 expression levels [65]. As shown in Table 4, the apparent affinity constants (*K*_D2_) for bivalent complexes of all the tested mAbs were in the low nanomolar range (≤1 nM), between 4.2-fold (mAb 3_7) and 8.6-fold (mAb 10_3) lower than that of the wild type IgG (*K*_D2_ = 1.2 nM). As already observed for monovalent binding, association rate constants (*k*_a_) were all very similar to one another (Table 4), with small increments with respect to the association rate constants (*k*_a_) measured for monovalent binding (about 2-fold for wild type IgG and 1.3-fold for all the mutated mAbs). On the contrary, the dissociation rate constants (*k*_d_) were significantly lower compared with the dissociation rate constants (*k*_d_) measured for monovalent binding (about 125-fold for wild type IgG and 209 and 295-fold for 3_7 and 10_3 mAbs, respectively). *k*_d_ values were between 4.4-fold (mAb 3_7) and 8.6-fold (mAb 10_3) lower than that of the wild type IgG (*k*_d_ = 1.28 × 10^−3^ 1/s), as already observed for monovalent binding, thus indicating that all the mutated CDR3s give rise to a more stable binding to PD-L1.

### 3.5. Binding Assays of the Affinity Matured IgGs to hPBMCs and Effects on T Cell Function

As the sequence 10_3 was the best binder of all both as the scFv format and as the full length antibody, we further investigated its binding properties to PD_L1 expressed in its native conformation on the plasma membrane and evaluated whether this binding had any implications on T cell function. To this aim, binding curves were performed on hPBMCs from healthy donors, firstly activated with anti-CD3/CD28 beads to stimulate the PD-L1 expression and then bound to a wide range of IgG concentrations.

**Figure 5:**
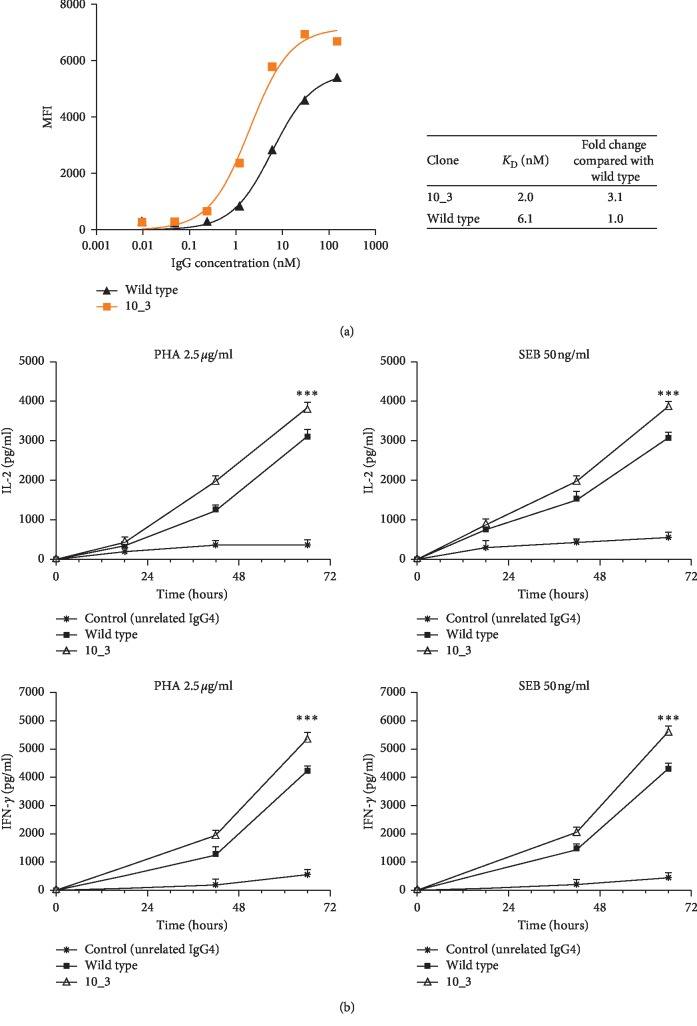
Binding and biological activity of the affinity matured mAb 10_3 on human lymphocytes. (a) The graph shows the binding curve of the selected mAb 10_3 to activated hPBMCs expressing PD-L1, in comparison with the wild type IgG. The binding was evaluated by flow cytometry, and the MFI of the CD2-positive cells bound to the IgGs was plotted against IgG concentration. The apparent affinity constant of the novel mAb 10_3 and its fold increase are reported. (b) The curves represents the effects of the affinity matured anti-PD-L1 mAb 10_3 on cytokine secretion of T cells after stimulation. hPBMCs were stimulated with PHA (2.5 *μ*g/mL) or SEB (50 ng/mL) in the absence or in the presence of the wild type antibody or its derived antibody 10_3. IL-2 and IFN-*γ* levels were measured in the supernatants by ELISA at different timepoints. An unrelated antibody was used as a negative control. Cytokine levels were reported as the mean of at least two determinations obtained in two independent experiments. Error bars indicate mean ± SD. *p* values for the cytokine concentration obtained with mAb 10_3 relative to the wild type mAb: ^*∗∗∗*^*p≤*0.001.

As indicated in Figure 5(a), mAb 10_3 bound to hPBMCs with a threefold higher affinity than the wild type IgG, showing a subnanomolar apparent affinity constant. Moreover, mAb 10_3 bound only to activated lymphocytes, thus confirming that the mutagenesis had not changed its binding specificity for PD-L1 (Supplementary [Supplementary-material supplementary-material-1]).

To test whether the novel generated variant 10_3 acquired better biological activity than the parental mAb, we tested mAb 10_3 in a T cell cytokine secretion assay [66] in comparison with the wild type mAb. As shown in Figure 5(b), the affinity matured antibody was able to induce the secretion of both IL-2 and IFN-*γ* by hPBMCs, stimulated with either PHA or SEB, more efficiently than the parental mAb, thus suggesting that the increase in the affinity has indeed potentiated also the biological properties of the parental mAb.

## 4. Discussion

Monoclonal antibodies have proven to be versatile therapeutic tools for the treatment of several disorders [1–4] and because of their potentials, such as the extreme target selectivity, their discovery is in constant development [5, 6]. The antibody specificity is of main importance in cancer immunotherapy to avoid the side effects of conventional chemotherapies. In this field, besides the classical targeting of tumor antigens [29], antibodies are also widely used as immune checkpoint inhibitors, aiming at the reactivation of the antitumor immune response [45–59]. Despite the success achieved with this kind of therapy, many cases of resistance or relapse after treatment with the clinically approved checkpoint inhibitors (anti‐CTLA-4, anti‐PD-1 and anti-PD-L1) are still reported [60, 61], thus explaining the efforts of discovering other checkpoint inhibitors for possible combination treatments.

Along with the identification of novel lead antibodies, many progresses have been done in antibody engineering, in order to improve the therapeutic efficacy and safety profile [7, 8]. In particular, the improvement of the antibody affinity is crucial to increase efficacy and enhance clinical outcomes, and it is achieved by means of *in vitro* technologies that allow to reach picomolar affinities [14, 24, 26]. Yeast surface display (YSD) is the most widely used and powerful approach, taking advantage from an efficient library generation by *in vivo* yeast recombination and from the FACS-based isolation of improved antibody variants [24, 26]. In this study, we described an optimization of the YSD methodology, focusing on the setup of a faster and more effective protocol for the generation of novel high affinity antibodies. In the scenario of the discovery of novel checkpoint inhibitors, our work aimed to identify high affinity antibodies targeting PD-L1, which is overexpressed in several kinds of human tumors, making them resistant to T cell-mediated killing [40–42].

We affinity-matured the sequence of a novel anti-PD-L1 antibody (named “PD-L1_1”), identified in our lab by phage display technology and tested *in vitro* and *in vivo* for its antitumor activity [62]. We used a CDR-targeted mutagenesis protocol involving a single CDR, that is, the heavy chain variable region CDR3, mainly because it is well known that CDR3s generally make more extensive contacts than the other CDRs in the antibody-antigen interactions [67, 68].

In order to make the selection of high affinity variants easier and faster, we generated high quality libraries, where at least 50% of the sequences carried missense mutations, thus avoiding the presence of too many wild type sequences, which could potentially interfere with the selection of mutated antibodies.

We had previously demonstrated that, for very short sequences, like CDRs, it was not possible to increase the mutation frequency up to a desired value by a single error-prone PCR reaction just decreasing the template amount, which inversely correlates with the mutation rate (unpublished data). For this reason, we performed 10 sequential cycles of error-prone mutagenesis of the CDR3. As a proof of the effectiveness of this approach, we carried out a systematic and extensive HTS analysis of the repertoires of CDR3s generated by the 10 rounds of random mutagenesis and analyzed them both at nucleotide and amino acid levels. By this method, the percentage of reads with the wild type sequence decreased in a nonlinear fashion, down to 5% at round 10, which corresponded to 10% wild type translated sequences. About 5–10% more amino acid wild type reads than nucleotide ones were found at each round and represented the fraction of sequences that contained only silent mutations.

Besides showing the least wild type frequency, the pool of fragments generated at cycle 10 was also the most heterogeneous in terms of number of mutations per sequence, as demonstrated by the comparable fractions of fragments containing 1 and 2 amino acid mutations (about 28% of sequences carrying missense mutations), in addition to a good percentage of sequences with 3 mutations (15%). Considering that the CDR3 consisted only in 11 amino acids, it was not desirable to have too many mutations per sequence, which could have a negative impact on antigen binding. Sequences containing more than 4 mutations were 20%, but this percentage was not so different from those observed in the previous rounds. Sequences containing stop codons were 20% of the total sequences, but the method used to analyze and select the yeast-antigen complexes easily excluded them from the selection because the C-term of the scFvs was labeled, thus detecting only the full length proteins.

Although the CDR3 pool of mutants obtained after 10 mutagenesis rounds had the desired features, we also generated a library using fragments from mutagenesis cycle 3 (library 3), thus testing whether 50% of sequences with missense mutations was sufficient for our purposes, instead of performing 10 cycles (library 10).

As it was very difficult to extract high-quality yeast plasmid DNA for HTS applications, we did not carry out the same HTS analysis of the full length scFvs after yeast recombination; we reasonably hypothesized that the yeast-scFv libraries should reflect almost the same features as the pools of CDRs which they derived from. Consequently, considering that the theoretical complexities of libraries 3 and 10 were 2 × 10^8^ and 1.7 × 10^8^, respectively, more or less 1 × 10^8^ and 1.2 × 10^8^ clones should be “good” clones (50% and 70%, respectively). These were considered good starting complexities to ensure an efficient and successful isolation of high affinity variants [24, 26].

According to the different features of the two libraries, we used slightly different selection schemes, which shared essentially a common strategy, i.e., the use of saturation concentrations of the dimeric rhPD-L1-Fc antigen (30 or 10 nM) in the first FACS selection steps (1 or 2 steps), followed by a fast drop of this concentration (10 pM) in the last cycle. 10 pM was the concentration which did not result in a detectable binding of the wild type yeast. Such a strategy substantially differs from the others so far reported in the literature, where the concentrations of the soluble antigen gradually decrease at each selection round [24]. In this way, very few clones were isolated (only 75 clones from library 3 and 900 from library 10) at the expense of the diversity of the selected population, but avoiding the selection of the wild type clones and increasing the chance to catch the top binders only. In fact, despite the very small sampling of sorted clones (24 clones randomly chosen from each sorted library), we did not find any wild type scFv among the sorted yeasts from library 10, whereas fewer wild type clones were found in sorted library 3 than in the parental library (25% *versus* 42%). This reasonably reflected the lower wild type starting frequency of library 10 compared to library 3 (10% *versus* 43%) and the higher sorting gate stringency used since the first step of library 10 selection.

By this small-scale sequencing analysis, 12 different mutated clones were identified overall. As a proof of the effectiveness of this selection approach, we confirmed that half of them (clones 3_3, 3_7, 3_14 and 3_17 from library 3 and clones 10_3 and 10_12 from library 10) really bound to PD-L1 limiting concentration (10 pM) better than the wild type yeast clone, showing a 6.3- to 9.8-fold improvement of their apparent affinity constants in binding curves to dimeric rhPD-L1-Fc. Interestingly, all the four clones from library 3 were double mutants, carrying the same mutation in the first CDR3 amino acid (Tyr turned into Ser) plus another one in the tenth position (Asp turned into Gly, Asn, Ala or Tyr), whereas the two clones from library 10 were triple mutants, which shared two of the three amino acid substitutions with the double mutant clone 3_14. Although some of these clones showed a certain enrichment (7 copies for clone 3_14), the small sampling of sorted clones was not sufficient to highlight any correlation between their enrichment level and the entity of their affinity improvement. However, it is clear that the same mutation hotspots were brought to light, starting from libraries generated by a different number of mutagenesis rounds but with a common selection approach. This result suggests that such a platform could be useful for a rapid identification and screening of the key amino acid residues involved in the antigen binding and modulation of the binding strength. In addition, of main relevance is the observation that two of the best clones were present in the yeast population selected from both libraries (clones 3_3 and 3_14, corresponding to clones 10_4 and 10_2).

Surface plasmon resonance data related to the IgGs converted from the best scFvs were found to be in good agreement with apparent affinity constants obtained for the yeast-scFv clones, confirming IgGs 3_17 and 10_3 as the best of all the selected variants. In fact, all the IgGs showed lower equilibrium dissociation constants (*K*_D_) than the wild type one, in the formation of both monovalent complexes (from 49 to 84 nM for mutated IgGs *versus* 314 nM for the wild type) and bivalent ones (from 0.14 to 0.28 nM for mutated IgGs *versus* 1.2 nM for the wild type) with PD-L1, but the fold changes of their affinity compared to wild type were more or less the same in both cases. The 300- to 350-fold lower values of the *K*_D_ obtained for bivalent complexes, which mimic the physiological binding conditions, are likely to allow strong specific binding interactions of IgGs with cells expressing high PD-L1 levels compared to normal cells, thus limiting unwanted toxic reactions.

The significantly lower dissociation kinetics observed for the affinity matured IgGs in both kinds of complexes indicate a more stable binding, which could ensure a more effective treatment as a consequence of a prolonged exposure to the molecule. Although binding curves highlighted still weak transient interactions (Figures 4(b)–4(g)), it could be another advantage to reduce unwanted side reactions toward normal cells with low PD-L1 expression levels [69].

Despite the consistency between the *K*_D_ values obtained in different systems (yeast display and SPR), we observed that the PD-L1 format used for *K*_D_ determination influenced the magnitude of the *K*_D_ values. In fact, when monomeric protein was used, *K*_D_ values were about 2log higher (from 49 to 84 nM) than those estimated in presence of dimeric PD-L1, and this occurred in both yeast display (from 0.46 to 0.73 nM) and SPR studies (from 0.14 to 0.28 nM). We supposed that the presence of two near domains in dimeric PD-L1 strongly promotes the binding; in fact, once a monomer of the dimeric protein has bound to an IgG arm or to a scFv molecule expressed on the yeast surface, the binding of the second monomer is greatly facilitated. Hence, the affinity of the yeast-scFv or the whole IgG for PD-L1 is higher in these conditions (consequently with lower *K*_D_ values).

Although we observed *K*_D_ differences for each scFv/IgG according to the PD-L1 format used, the consistency between the *K*_D_ sets obtained with different formats let us reasonably hypothesize that we would have selected the same scFvs also in presence of the PD-L1 monomeric format.

The best of all IgGs (10_3) also gave good results in binding and functional assays performed on human PBMCs as a source of PD-L1. In fact, this IgG bound to PBMCs with higher affinity than the wild type IgG, although the magnitude of this increase (3-fold improvement) was not comparable to that observed in SPR kinetic studies. In our opinion, this difference could be mainly due to different PD-L1 formats used in the two assays. In fact, while rhPD-L1 used in SPR studies is a dimer (the same as that used for the yeast selection), native PD-L1 expressed on the plasma membrane is a monomer.

As a consequence of its affinity improvement, the antibody 10_3 was able to induce a stronger T cell stimulation than the wild type, as demonstrated by the increase in the levels of IL-2 and IFN-*γ* measured in the culture medium at different timepoints, in comparison with the wild type antibody.

All the binding data, related to both the scFvs and the converted IgGs, in addition to the functional T cell stimulation assay performed with the top binder IgG 10_3, strongly support the idea that the optimization of the yeast display platform, proposed in this work, could lead to a quick affinity maturation of novel antibodies, in just two selection steps by a fast drop in antigen concentration and starting from libraries containing almost half of sequences with missense mutations in a single CDR. Of course, higher starting rates of mutation increase the chance to select a more diversified and heterogeneous pool of sequences.

## 5. Conclusions

In this paper, we set up a fast antibody CDR affinity maturation protocol by an optimization of the yeast display technology. By a sequential error-prone mutagenesis of a single CDR, coupled with a novel and stringent FACS-based selection approach, we succeeded in isolating novel anti-PD-L1 scFvs with improved affinity compared to the wild type one. The evidence that the converted IgGs retained a comparable affinity improvement for PD-L1, showing subnanomolar affinities against native PD-L1, in addition to the demonstration of functional activity of the best affinity matured IgG, proved the effectiveness of this novel affinity maturation strategy. Thanks to their kinetic properties, these novel antibody variants could be good candidates for a PD-L1-targeted cancer immunotherapy. Further *in vivo* experiments are needed to assess the potency of the novel mAbs in inducing the regression/remission of the tumor.

## Figures and Tables

**Table 1:** Sequences of clones from sorted library 3^a^.

CDR3 position^b^	1	2	3	4	5	6	7	8	9	10	11
Wild type	T	K	W	E	L	V	D	P	Y	D	Y
3_1	**A**	K	W	E	L	V	D	P	Y	D	Y
3_2	**S**	K	W	E	L	V	D	P	Y	D	Y
3_3	**S**	K	W	E	L	V	D	P	Y	**N**	Y
3_4	T	K	W	E	L	V	D	P	Y	**Y**	Y
3_5	**S**	K	W	E	L	V	D	P	Y	**G**	Y
3_6	**S**	K	W	E	L	V	D	P	Y	D	Y
3_7	**S**	K	W	E	L	V	D	P	Y	**Y**	Y
3_8	**S**	K	W	E	L	V	D	P	Y	D	Y
3_9	**S**	K	W	E	L	V	D	P	Y	**G**	Y
3_10	**S**	K	W	E	L	V	D	P	Y	**G**	Y
3_11	T	K	W	E	L	V	D	P	Y	D	Y
3_12	T	K	W	E	L	V	D	P	Y	D	Y
3_13	**S**	K	W	E	L	V	D	P	Y	**G**	Y
3_14	**S**	K	W	E	L	V	D	P	Y	**G**	Y
3_15	**S**	K	W	E	L	V	D	P	Y	**N**	Y
3_16	T	K	W	E	L	V	D	P	Y	D	Y
3_17	**S**	K	W	E	L	V	D	P	Y	**A**	Y
3_18	**S**	K	W	E	L	V	D	P	Y	D	Y
3_19	T	K	W	E	L	V	D	P	Y	D	Y
3_20	**S**	K	W	E	L	V	D	P	Y	**G**	Y
3_21	T	K	W	E	L	V	D	P	Y	D	Y
3_22	T	K	W	E	L	V	D	P	Y	D	Y
3_23	**S**	K	W	E	L	V	D	P	Y	**A**	Y
3_24	**S**	K	W	E	L	V	D	P	Y	**G**	Y

^a^Each clone is identified with the library number which it belongs to, followed by a progressive number. ^b^The numbers in the first row correspond to the position of the amino acids in the CDR3. The mutations are indicated in bold.

**Table 2:** Sequences of clones from sorted library 10^a^.

CDR3 position^b^	1	2	3	4	5	6	7	8	9	10	11
Wild type	T	K	W	E	L	V	D	P	Y	D	Y
10_1	T	K	W	E	L	V	D	P	**F**	D	Y
10_2	**S**	K	W	E	L	V	D	P	Y	**G**	Y
10_3	**S**	K	W	E	L	V	D	P	**F**	**G**	Y
10_4	**S**	K	W	E	L	V	D	P	Y	**N**	Y
10_5	**S**	K	W	E	L	V	D	P	Y	**N**	Y
10_6	**S**	K	W	E	L	V	D	P	Y	**G**	Y
10_7	**S**	K	W	E	L	V	D	P	Y	**G**	Y
10_8	T	K	W	E	L	V	D	P	**F**	**N**	Y
10_9	**S**	K	W	E	L	V	D	P	Y	**G**	Y
10_10	**S**	K	W	E	L	V	D	P	Y	**G**	Y
10_11	**S**	K	W	E	L	V	D	P	Y	**N**	Y
10_12	**S**	K	W	E	L	V	D	P	Y	**G**	N
10_13	T	K	W	E	L	V	D	P	**F**	**N**	Y
10_14	T	K	W	E	L	V	D	P	**F**	**G**	Y
10_15	**S**	K	W	E	L	V	D	P	Y	**N**	Y
10_16	**S**	K	W	E	L	V	D	P	Y	**G**	Y
10_17	T	K	W	E	L	V	D	P	**F**	D	Y
10_18	**S**	K	W	E	L	V	D	P	Y	**N**	Y
10_19	**S**	K	W	E	L	V	D	P	Y	**G**	Y
10_20	**S**	K	W	E	L	V	D	P	Y	**G**	Y
10_21	**S**	K	W	E	L	V	D	P	Y	**N**	Y
10_22	**S**	K	W	E	L	V	D	P	Y	**G**	Y
10_23	**S**	K	W	E	L	V	D	P	**F**	**G**	Y
10_24	**S**	K	W	E	L	V	D	P	**F**	**G**	Y

^a^Each clone is identified with the library number which it belongs to, followed by a progressive number. ^b^The numbers in the first row correspond to the position of the amino acids in the CDR3. The mutations are indicated in bold.

**Table 3:** Affinity and rate constants of rhPD-L1-his monovalent binding to anti-PD-L1 IgGs.

mAb	Kinetic analyses^a^				Steady-state analyses^b^
	*k*_a_ (1/Ms)^a^	*k*_d_ (1/s)^a^	*K*_D_ (nM)^a^	*K*_D_ fold change	*K*_D1_ (nM)^b^
Wild type	(5.12 ± 0.72) × 10^5^	(1.61 ± 0.30) × 10^−1^	314 ± 43.3	1	378 ± 61
**3_3**	(8.21 ± 0.57) × 10^5^	(4.68 ± 0.20) × 10^−2^	57.1 ± 4.67	5.49	69.3 ± 3.86
**3_7**	(7.09 ± 0.69) × 10^5^	(6.01 ± 0.06) × 10^−2^	84.8 ± 7.71	3.73	101 ± 4.57
**3_14**	(7.88 ± 0.59) × 10^5^	(4.68 ± 0.21) × 10^−2^	59.6 ± 5.43	5.26	67.1 ± 5.77
**3_17**	(7.32 ± 0.33) × 10^5^	(3.58 ± 0.20) × 10^−2^	49.0 ± 2.21	6.4	61.1 ± 4.98
**10_3**	(8.35 ± 0.49) × 10^5^	(4.37 ± 0.30) × 10^−2^	52.3 ± 4.18	6.0	62.9 ± 6.07
**10_12**	(7.70 ± 0.76) × 10^5^	(6.30 ± 0.09) × 10^−2^	81.8 ± 7.73	3.83	88.5 ± 7.24

^a^Kinetic (*k*_a_ and *k*_d_) and equilibrium dissociation (*K*_D_) constants for monovalent complexes were calculated by fitting binding curves (Figures 4(a)–4(g)) to the 1 : 1 Langmuir binding model. The equilibrium dissociation constants (*K*_D_) were calculated from the relationship *K*_D_ = *k*_d_*/k*_a_. The reported constants are average values obtained from at least four independent analyses using different biosensors, sample preparations, ligand densities and analyte concentration gradients on the flow cell surfaces. Data are reported with standard deviation. *K*_D_ fold changes compared to wild type are reported. ^b^Equilibrium binding analyses. Equilibrium dissociation constants (*K*_D1_) were calculated by non‐linear curve fitting of 1 : 1 Langmuir binding isotherms by plotting equilibrium response toward analyte concentrations (data not shown). The average values are reported with standard deviation.

**Table 4:** Apparent affinity and rate constants of anti-PD-L1 IgG bivalent binding to rhPD-L1-Fc.

mAb	*k*_a_ (1/Ms)^a^	*k*_d_ (1/s)^a^	*K*_D2_ (nM)^a^	*K*_D2_ fold change
Wild type	(1.09 ± 0.19) × 10^6^	(1.28 ± 0.16) × 10^−3^	1.2 ± 0.22	1
**3_3**	(1.14 ± 0.20) × 10^6^	(1.93 ± 0.38) × 10^−4^	0.174 ± 0.048	6.89
**3_7**	(1.03 ± 0.21) × 10^6^	(2.87 ± 0.53) × 10^−4^	0.287 ± 0.079	4.18
**3_14**	(1.05 ± 0.20) × 10^6^	(1.76 ± 0.39) × 10^−4^	0.172 ± 0.049	6.97
**3_17**	(1.01 ± 0.20) × 10^6^	(1.59 ± 0.17) × 10^−4^	0.165 ± 0.050	7.27
**10_3**	(1.10 ± 0.23) × 10^6^	(1.48 ± 0.34) × 10^−4^	0.139 ± 0.043	8.63
**10_12**	(1.07 ± 0.20) × 10^6^	(2.32 ± 0.39) × 10^−4^	0.223 ± 0.058	5.38

^a^Kinetic (*k*_a_ and *k*_d_) and apparent affinity (*K*_D2_) constants for bivalent complexes were calculated by fitting binding curves (Figures 4(h)–4(n)) to the 1 : 1 Langmuir binding model. The apparent affinity constants (*K*_D2_) were calculated from the relationship *K*_D_ = *k*_d_*/k*_a_. The reported constants are average values obtained from at least three independent analyses using different biosensors, sample preparations, ligand densities and analyte concentration gradients on the flow cell surfaces. Data are reported with standard deviation. *K*_D2_ fold changes compared to wild type are reported.

## Data Availability

No data were used to support this study.

## Abbreviations

BSA: Albumin from bovine serum
CFU: Colony-forming units
CTLA-4: Cytotoxic T lymphocyte-associated antigen 4
FACS: Fluorescence-activated cell sorting
FBS: Fetal bovine serum
FDA: Food and Drug Administration
HCDR3: Heavy chain complementarity determining region 3
hPBMCs: Human peripheral blood mononuclear cells
HTS: High-throughput sequencing
ICEB: Ice-cold electroporation buffer
IFN-*γ*: Interferon-*γ*
IgG: Immunoglobulin G
IL-2: Interleukin 2
*K*_D_: Equilibrium dissociation constant
*k*_a_: Association rate constant
*k*_d_: Dissociation rate constant
LAD: Lithium acetate-dithiothreitol
mAbs: Monoclonal antibodies
NSCLC: Non-small cell lung cancer
OD: Optical density
PBS: Phosphate buffered saline
PD-1: Programmed death-1
PD-L1: Programmed death ligand-1
RCC: Renal cell carcinoma
scFv: Single chain variable fragment
SDS-PAGE: Sodium dodecyl sulfate-polyacrylamide gel electrophoresis
SPR: Surface plasmon resonance
VH: Heavy chain variable region
VL: Light chain variable region
YNB: Yeast Nitrogen Base
YPD: Yeast-Peptone-Dextrose
YSD: Yeast surface display.

## Supplementary Materials

Supplementary Materials Figure S1 is a supplementary table to Materials and Methods, in which all the culture media used for yeast cultures are listed, in addition to all the reagents and indications necessary for their preparation. Figure S2 provides additional information to Section 3.5. It shows the flow cytometry analysis of the binding specificity of the best affinity matured antibody to PD-L1, in comparison to the wild type antibody.
