# Supplementary material for: Rapid Affinity Maturation of Novel Anti-PD-L1 Antibodies by a Fast Drop of the Antigen Concentration and FACS Selection of Yeast Libraries
Source: Biomed Res Int. 2019 Dec 28;2019:6051870. doi: 10.1155/2019/6051870 (PMC6959147; doi:10.1155/2019/6051870)
Supplement: Supplementary Materials — Figure S1 is a supplementary table to Materials and Methods, in which all the culture media used for yeast cultures are listed, in addition to all the reagents and indications necessary for their preparation. Figure S2 provides additional information to Section 3.5. It shows the flow cytometry analysis of the binding specificity of the best affinity matured antibody to PD-L1, in comparison to the wild type antibody. [file 6051870.f1.pdf]

Supplementary materials

yeast culture media and solutions	composition	supplier	notes
YPD	deionized water		autoclaving at 121°C for 20’
	10g/L yeast extract	sigma	
	20g/L peptone	sigma	
	20g/L agar (only for semisolid medium)	sigma	
	2% D-glucose		freshly added after autoclaving
	100U/ml penicillin and 100 µg/ml streptomicin	gibco	
selective medium YNB	deionized water		autoclaving at 121°C for 20’
	6.8 g/L yeast nitrogen base	sigma	
	20g/L agar (only for semisolid medium)	sigma	
	2% D-glucose		freshly added after autoclaving
	1X yeast synthetic drop out without tryptophan		
	100U/ml penicillin and100 µg/ml streptomicin	gibco	
induction medium	deionized water		autoclaving at 121°C for 20’
	6.8 g/L yeast nitrogen base	sigma	
	0.05% D-glucose		freshly added after autoclaving
	2% D-galactose		
	1X yeast synthetic drop out without tryptophan		
	100U/ml penicillin and100 µg/ml streptomicin	gibco	
20% D-glucose stock solution	deionized water		0.22 µm filtration
	200g/L D-glucose	sigma	
20% D-galactose stock solution	deionized water		0.22 µm filtration
	200g/L D-galactose	sigma	
10X yeast synthetic drop out without tryptophan stock solution	deionized water		0.22 µm filtration
	19.2g/L yeast synthetic drop out without tryptophan	sigma	

Figure S1: List and recipes of media and solutions used for yeast cultures.

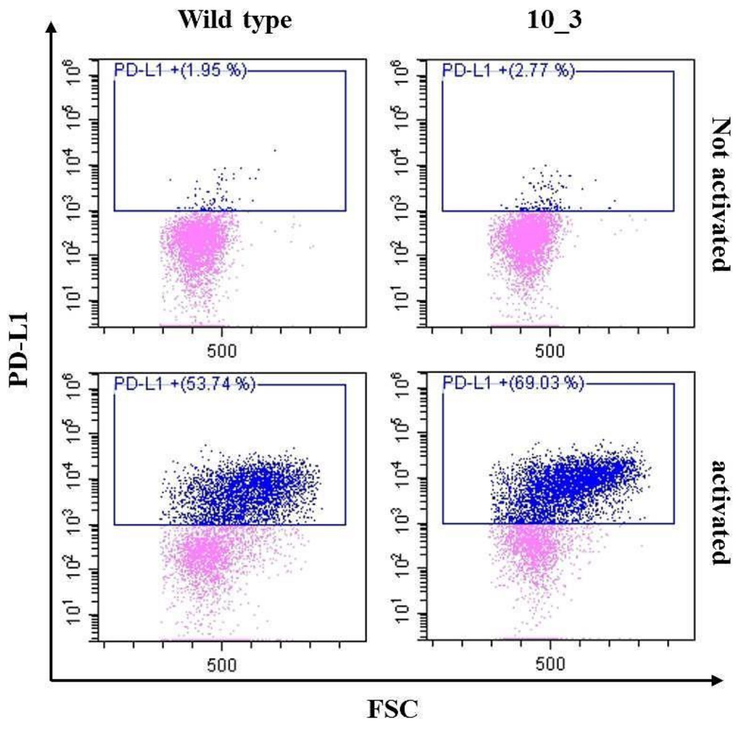

Figure S2: Validation of binding specificity of mAb 10_3 to activated hPBMCs. The panels show the percentages of not activated (upper panels) and activated (lower panels) lymphocytes (CD2-positive cells) which bound to a saturating concentration of the wild type and affinity matured mAb 10_3 (blue cells). The affinity maturation did not change the specificity for PD-L1, as demonstrated by the ability of binding only to activated cells.
